# Supplementary material for: Multilevel Social Determinants of Patient-Reported Outcomes in Young Survivors of Childhood Cancer
Source: Cancers (Basel). 2024 Apr 25;16(9):1661. doi: 10.3390/cancers16091661 (PMC11083567; doi:10.3390/cancers16091661)
Supplement: Supplementary file 1 [file cancers-16-01661-s001.zip › cancers-2966415-supplementary.pdf]

Supplementary Tables and Figures

Supplementary Figure S1. Flow diagram of study participants enrolled in this study.

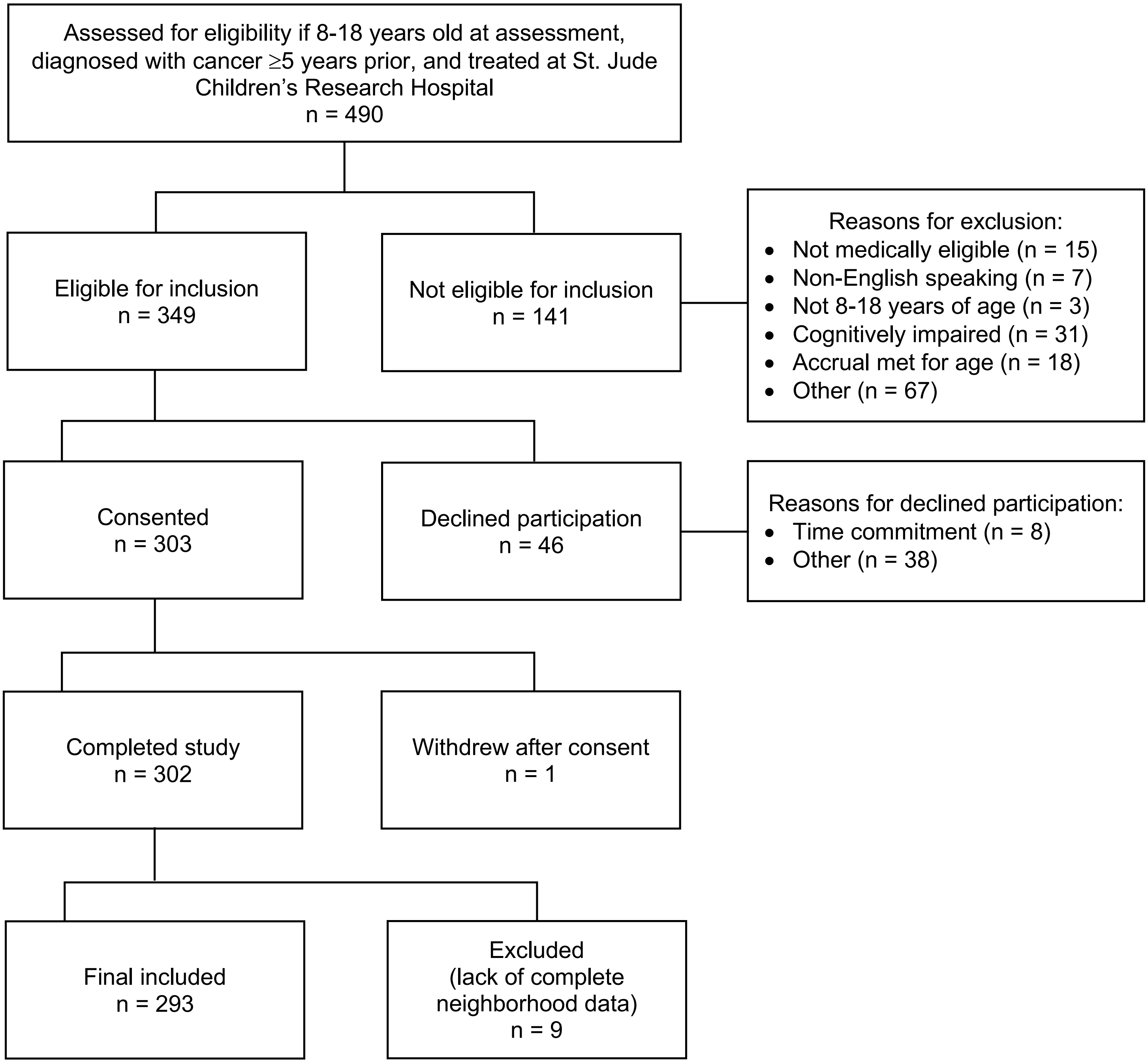

Supplementary Table S1. Deprivation status of all counties in the U.S. and the counties where survivors reside[†,‡].

Domains	All counties in the U.S. that were classified as deprivation (n=3,142)	Counties, where survivors resided, were classified as deprivation
	n (%)[§,#]	n (%)[§,#]
Socioeconomic status	1,372 (48.0)	160 (60.6)
Not completing high school	381 (12.3)	65 (22.9)
Unemployment	373 (11.7)	16 (5.6)
Children in poverty	480 (15.0)	38 (13.4)
Children in single-parent households	403 (12.6)	75 (26.4)
Social associations deprivation	307 (9.6)	32 (11.3)
Violent crime deprivation	415 (13.8)	114 (43.2)
Injury deaths deprivation	399 (13.8)	10 (3.5)
Physical environment	750 (23.5)	125 (44.0)
Severe housing problems	399 (12.5)	50 (17.6)
Driving alone to work	175 (5.9)	46 (16.2)
Long commute time	528 (16.5)	52 (18.3)

† Data from the County Health Rankings & Roadmaps by the Robert Wood Johnson Foundation.
‡ Not all counties have information on all indicators from the County Health Rankings; the denominators may vary.
§ The status of each indicator is classified as “deprivation” if the indicator’s Z-score of this county is ranked below 1 standard deviation among all countries in the U.S.
# Each domain (socioeconomic status and physical environment, respectively) of each county is classified as “deprivation” if one or more indicators of the respective domain is classified as deprivation.

Supplementary Table S2. Bivariate associations of contextual/social determinants with PROs among young survivors of childhood cancer.

Factors	Depression†	Psychological stress†	Fatigue†	Sleep disturbance †	Positive affect‡	Mobility‡
	B (95% CI)	B (95% CI)	B (95% CI)	B (95% CI)	B (95% CI)	B (95% CI)
Survivor factors (personal level)						
Age	0.14 (-0.22, 0.50)	0.54 (0.15, 0.92)**	0.06 (-0.40, 0.52)	0.36 (-0.02, 0.74)	-0.49 (-0.83, -0.14)**	-0.04 (-0.34, 0.26)
Time since diagnosis	-0.07 (-0.44, 0.29)	0.34 (-0.04, 0.73)	-0.04 (-0.49, 0.42)	0.15 (-0.22, 0.53)	-0.3 (-0.66, 0.05)	0.26 (-0.04, 0.56)
Sex						
Male	Ref	Ref	Ref	Ref	Ref	Ref
Female	3.8 (1.81, 5.85)***	4.58 (2.41, 6.75)***	2.3 (-0.28, 4.89)	1.16 (-1.02, 3.27)	-0.4 (-0.24, 1.62)	-0.32 (-2.03, 1.39)
Cancer Diagnosis						
Hematologic cancers	Ref	Ref	Ref	Ref	Ref	Ref
CNS tumors	0.21 (-3.08, 3.51)	0.17 (-3.35, 3.69)	3.64 (-0.44, 7.71)	-2.11 (-5.50, 1.29)	0.56 (-2.64, 3.76)	-3.98 (-6.64, -1.33)**
Solid tumors	-0.85 (-3.09, 1.40)	-1.52 (-3.94, 0.90)	-0.95 (-3.75, 1.86)	-0.46 (-2.34, 1.41)	-0.46 (-2.34, 1.41)	-0.46 (-2.34, 1.41)
Caregiver and family factors (family level)						
Annual household income						
≥ $75,000	Ref	Ref	Ref	Ref	Ref	Ref
<75,000	1.35 (-0.74, 3.40)	-1.13 (-3.39, 1.13)	0.63 (-2.01, 3.26)	0.37 (-1.81, 2.55)	-0.98 (-3.03, 1.06)	-0.32 (-2.05, 1.41)
# of caregiver chronic diseases	0.81 (0.02, 1.59)*	0.82 (-0.02, 1.67)	0.87 (-0.11, 1.85)	0.38 (-0.44, 1.19)	-0.31 (-1.08, 0.45)	-0.49 (-1.14, 0.15)
Family dynamics						
Cohesion	[illegible].11 (-0.21, -0.003)*	-0.06 (-0.16, 0.05)	-0.03 (-0.16, 0.10)	-0.09 (-0.19, 0.02)	0.03 (-0.06, 0.13)	0.10 (0.02, 0.18)**
Conflict	0.10 (-0.01, 0.22)	0.11 (-0.01, 0.23)	0.09 (-0.06, 0.23)	0.21 (0.09, 0.32)**	-0.12 (-0.23, -0.01)**	-0.09 (-0.18, 0.01)
Expressiveness	-0.01 (-0.11, 0.09)	-0.02 (-0.13, 0.09)	-0.06 (-0.19, 0.07)	-0.05 (-0.15, 0.06)	0.03 (-0.06, 0.13)	0.002 (-0.08, 0.09)
Caregiver PROs						
Anxiety	0.23 (0.12, 0.34)**	0.22 (0.10, 0.34)***	0.31 (0.18, 0.46)***	0.29 (0.18, 0.41)***	-0.16 (-0.26, -0.04)**	-0.11 (-0.21, -0.02)*
Fatigue	0.17 (0.05, 0.29)**	0.22 (0.10, 0.35)**	0.33 (0.18, 0.47)**	0.24 (0.12.0.36)**	-0.13 (-0.25, -0.01)*	-0.15 (-0.24, -0.05)**
Sleep disturbance	0.08 (0.04, 0.31)*	0.17 (0.02, -0.31)*	0.2 (0.13, 0.46)**	0.23 (0.09, 0.36)**	-0.06 (-0.19, 0.07)	-0.19 (-0.30, -0.08)***

Pain intensity	0.08 (-0.06, 0.21)	-0.03 (-0.17, 0.11)	0.13 (-0.03, 0.29)	0.09 (-0.05, 0.22)	-0.01 (-0.14, 0.12)	-0.05 (-0.16, 0.06)
Physical function	-0.05 (-0.22, 0.12)	-0.04 (-0.21, 0.13)	-0.18 (-0.38, 0.02)	-0.07 (-0.24, 0.10)	0.07 (-0.09, 0.23)	0.09 (-0.04, 0.22)
Neighborhood factors (county level)						
Socioeconomic status	1.50 (-0.69, 0.99)	-0.36 (-1.26, 0.55)	0.38 (0.67, 1.42)	1.14 (0.28, 2.00)**	0.25 (-0.57, 1.07)	-0.13 (-0.81, 0.56)
Physical environment	-0.81 (-2.44, 0.82)	2.59 (0.85, 4.32)**	1.75 (-0.29, 3.78)	1.74 (0.06, 3.41)*	-2.14 (-3.71, -0.56)**	-1.43 (-2.76, -0.10)*

Abbreviation: PROs, patient-reported outcomes; B, regression coefficient; CI, confidence interval; Ref, reference group

**P*<0.05, ***P*<0.01, ****P*<0.001

† Higher score indicates poorer PROs

‡ Higher score indicates better PROs
